# Security Architecture for Secure Train Control and Monitoring System

**DOI:** 10.3390/s23031341

**Published:** 2023-01-25

**Authors:** Yudha Purwanto, Muhammad Faris Ruriawan, Andry Alamsyah, Febry Pandu Wijaya, Dewi Nala Husna, Agri Kridanto, Fifin Nugroho, Anang Fakhrudin, Mu’ammar Itqon, Mochamad Yudha Febrianta, Sri Widiyanesti, Fussy Mentari, Alfian Akbar Gozali, Ade Romadhony

**Affiliations:** 1 School of Electrical Engineering, Telkom University, Jl. Telekomunikasi No. 1, Bandung 40257, Indonesia; 2 School of Economics and Business, Telkom University, Jl. Telekomunikasi No. 1, Bandung 40257, Indonesia; 3 Indonesian Rolling Stock Industry, Jl. Yos Sudarso No. 71, Madiun 63122, Indonesia; 4 School of Applied Science, Telkom University, Jl. Telekomunikasi, Bandung 40257, Indonesia

**Keywords:** train control, monitoring system, railway security, cryptography, system architecture

## Abstract

A Train Control and Monitoring System (TCMS) is a vital part of monitoring sensors in a train. The data output of sensors is sent wirelessly to the data server for monitoring. However, as the wireless channel used to send the data is a shared public network, the transmitted data are prone to hackers and attacks. This paper proposes the Securebox architecture to manage secure data transfer from the onboard Vehicle Control Unit (VCU) to the data server in TCMS. The architecture is comprised of four main functions: network management, buffer management, data management, and security management. The architecture has been successfully developed in an HSM (Hardware Security Modul) and verified using alpha and beta software testing to form a secure TCMS. From the real-time testing phase in an electric-diesel train, the average performance of the AES-based HSM showed 55% faster time processing with unnoticed 0.1% added memory usage compared to the 3DES. The secure TCMS also withstands MITM attack and provides end-to-end data security compared to the (Mobile Station) MS to Base Station (BS) only in GSM-R.

## 1. Introduction

In addition to traditional signaling for communication between trains and dispatchers, the railway system also requires an automated monitoring system to ensure the reliability of the moving railway system. The Train Control and Monitoring System (TCMS) is a system that could help manage the train by controlling, monitoring, and recording a number of train equipment and sensor activities. The modern TCMS has become a vital part of railway systems, as it provides train reliability and security [1]. To gain more capabilities and performance, the TCMS research has been conducted for real-time capability [2,3,4], signal control system [5], and security [6,7].

The vulnerability to cyberattacks on public transportation modes such as trains increases with the predetermined pattern of mobilization and the large number of people involved. The communication systems and command control mechanisms in TCMS are prone to attacks. A secure TCMS system is vital to ensure quality of service is delivered [2]. TCMS security can be in the form of physical, data, and network security. Our physical security research aims to develop a protection system from physical attacks such as network tapping, personnel assault, physical authorization techniques, and installed hardware. Data and network security aim to secure the communication between the onboard train control system and the data center.

Reflecting on a case of car hacking [8], a car that has an electrical control unit (ECU) can be exploited remotely, which results in the physical system being affected, such as steering and braking. This can happen in TCMS, where data from the vehicle control unit are sent over a public network prone to attack. In a public network, there is a vulnerability where anyone can enter the network, and further enter the TCMS system illegally in a man-in-the-middle (MITM) attack, which can cause train collisions [6]. These data are critical because they may not only provide information about the operation but also about the control mechanism of the train.

In a train-to-train and train-to-ground Communications-based Train Control (CBTC) network, Hardware Security Modules (HSMs) can be installed to secure client-server communication [9,10]. These devices help guarantee the confidentiality, integrity, and authenticity of the data by providing a cryptography function. The state-of-the-art research in [7] proved that data and network security are vital in the TCMS. The need for higher security standards was implemented using cipher block chaining-message authentication code (CBC-MAC) based on the 3DES algorithm for the European Rail Traffic Management System (ERTMS). However, based on the research in [11,12,13], the DES, 3DES, and A5/1 algorithm used in ERTMS have a security issue related to the weak keys. The use of the Global System for Mobile Communications for Railway (GSM-R) does not provide an end-to-end data protection, as it only encrypts data from the Mobile Station (MS) to the Base Station (BS) and uses one-way authentication from MS to BS only [14]. This vulnerability may result in an unsecured communication problem, especially in a Man in The Middle (MITM) attack. The lack of information on the standard security architecture for TCMS is also a vital problem for ensuring interoperability between devices.

In this study, the TCMS security architecture was designed in a Securebox architecture and implemented in Securebox HSMs. The architecture is composed of four main functions: network management, buffer management, data management, and security management. The network management is responsible for successful communication between the onboard train system and the data server. Buffer management is accountable for data integrity, which acts as a buffer for message synchronization. The data management is responsible for the synchronization and data parsing function, and the security management is responsible for data confidentiality. The architecture was developed in an x86 computer platform that implemented AES and 3DES cryptography algorithm modes to provide end-to-end data security. The AES algorithm was chosen as it provides a better security performance than 3DES [11].

The research contribution in the form of a Securebox architecture for secure TCMS was achieved. The Securebox architecture has been successfully developed in a Securebox HSMs, verified, and validated according to the design criteria in a laboratory environment. Furthermore, the HSMs were also implemented and analyzed in an electric-diesel train prototype at the Indonesian Rolling Stock Industry, a railway manufacturing industry in Indonesia. An AES-based HSM showed 55% faster time processing with unnoticed 0.1% added memory usage compared to the previous 3DES proposal. It protected the TCMS from the risk of repetition, insertion, corruption, and masquerade data from a MITM attack. It also provided end-to-end data protection compared to the use of GSM-R, which only encrypts data from MS to BS.

In this paper, the related issues will be elaborated on and discussed. In Section 2, the advanced research of TCMS security is summarized based on the inherent characteristics. The novelty of this research is discussed in Section 3, which covers the proposed architecture, HSM design, and the Secure TCMS setup and validation processes. In Section 4, the output of the secure TCMS is discussed in a performance and security analysis. Based on the analytical process in Section 4, the conclusion of the research is shown in Section 5. It also suggests for further research in our research roadmap.

## 2. Related Work

The reliability of data transmission in real-time communication has been studied in several areas. In railway communication, this aspect is also a vital part of monitoring the operation of a railway. Research in [1] designed a reliable, adaptable, and flexible railway control and management system while ensuring safety standards. The additional security system proposed to increase control over alerts and networks available on the entire system in this research. Research in [3] developed railway asset real-time monitoring, tracking, and tracing functions to increase reliability. The integrated system obtains data from sensors installed on the train. It also increased interoperability by enabling the use of recorded data in real-time and implemented the GNSS (Global Network Satellite System) system to track the location of the railway.

For the tracking and monitoring system function, integrated security of the railway control system has attracted the attention of researchers. Research in [15] proposed an integrated model which effectively analyzed all classes of attacks. The combination of the component-based approach, the semi-natural model, the simulation model, and the analytical model increased the security of critical infrastructure by improving the quality of attack actions analysis in multi-step attack scenarios. This model was then reported further in [16], which provided a rational solution based on functional requirements and non-functional limitations to the system. The combination of design, development, and verification techniques within a single approach can improve the semi-natural model of the railway infrastructure.

From the network point of view, an intrusion attack can also disturb and disrupt the Communications-based Train Control (CBTC) network. However, implementing a traditional anomaly-based intrusion detection system (IDS) is not enough, because the traditional IDS may produce a false positive alarm caused by a system fault. A further study was reported in [17], which proposed an intrusion detection model using a fusion of network and device states. The proposed method can identify the difference between the abnormality among anomalies caused by cyber-attacks and by system faults. This method can distinguish 97.64% of true abnormalities caused by cyber-attacks. A more holistic approach was proposed in [18], which introduced the S4R project as a risk and resilience assessment specific to railway networks. The S4R platform is an integrated platform that has predictive risk and resilience assessment, data processing, decision support, monitoring, and anomaly detection tools. This platform can effectively analyze all classes of attacks to improve the resilience of railway networks. Specific intra-vehicular communication using the Wi-Fi-based CBTC network security was proposed in [19]. This study proposed the use of a Host Identity Protocol (HIP) and a Balise Transmission Module (BTM) to withstand jamming attacks in the 802.11 CBTC network.

The security of data communication in a train is a vital part of real-time monitoring and tracking. From a passive eavesdropping attack, the attacker can attack further by MITM, which not only reads but also can modify or produce fake data. The cyber-physical vulnerability analysis in [6] found the attack can cause train collisions by manipulating the control message, such as the safety margin between trains. On the TCMS system, a MITM attack could happen in the wireless communication between the CBTC and the automatic train supervision (ATS), especially when using a User Datagram Protocol (UDP) format combined with knowledge of train signaling. In [9], the data security function was handled in Hardware Security Modules (HSMs). These devices play an important function in guaranteeing the confidentiality, integrity, and authenticity of the data by providing a cryptography function. A technical solution to secure the security function was introduced in [7], which conducted a cyber security analysis on the European train control system. The study looked at the European Rail Traffic Management System (ERTMS), which was designed in the 1990s, with the security considerations and strategies that existed at that time. ERTMS uses GSM-R communication technology and needs improvement. This study carried out a security test on the ERTMS safety layer related to the current security threats. These threats can be in the form of passive attacks and active attacks. Passive attacks are eavesdropping attacks. The solution was the use of higher security standards, one of which is using encryption algorithms. The algorithm used a cipher block chaining-message authentication code (CBC-MAC) based on the 3DES algorithm. Further research in [13] evaluated ERTMS vulnerability. It reported ERTMS was exposed by the exploitability of the A5/1 and 3DES algorithms. If the algorithm is compromised, then the attacker can read the message or even send injection attack.

From the cryptographic point of view, Refs. [11,12,13] showed some compromises in the 3DES algorithm compared to the AES algorithm. DES has security issues related to weak keys because it uses the same components for encryption and decryption. AES uses different parts between encryption and decryption, thereby reducing the possibility of weak keys. Based on tests, it was found that AES performance and computing cost was commensurate with DES but with better security features. Our research proposes a security architecture for secure TCMS, using HSMs that provide a more reliable cryptographic algorithm.

## 3. Proposed Architecture

**Figure 1:**
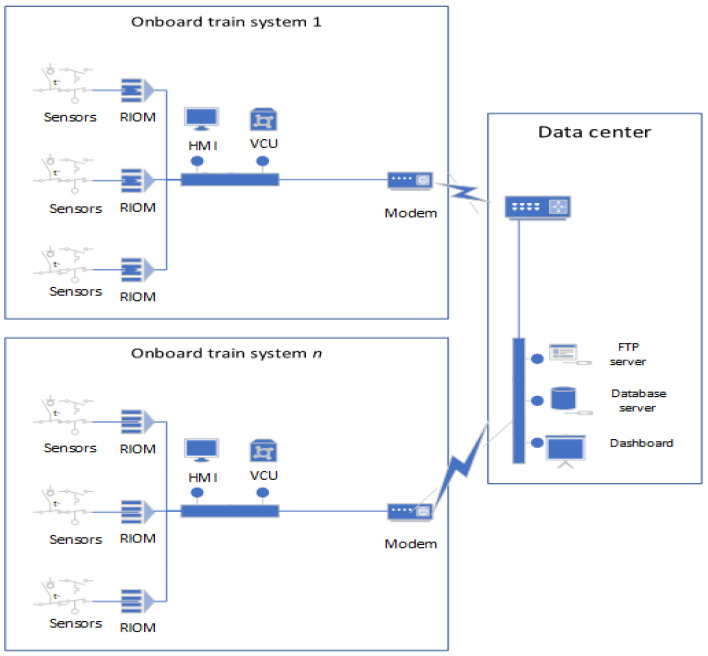
Existing TCMS model without security function.

The existing TCMS without a security function consists of an onboard system inside the trains, which communicate with a data center such as shown in Figure 1. The onboard train system is located inside a train and consists of sensors connected to a Remote Input Output Module (RIOM) and connected to a Vehicle Control Unit (VCU). The collected data from sensors in the VCU are then transmitted to the data server. This existing TCMS only implements a plaintext file transfer function and a monitoring function.

**Figure 2:**
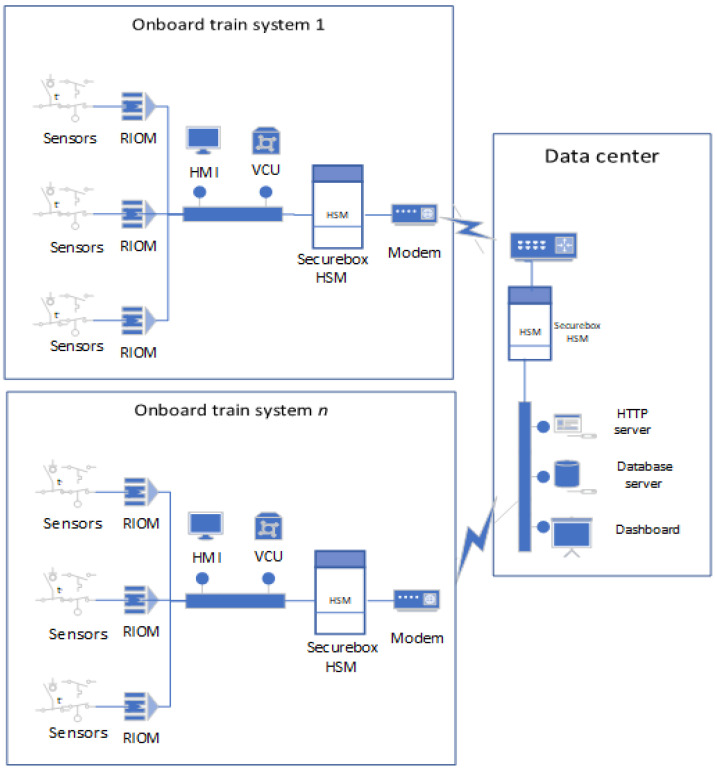
Secure TCMS Model.

In the secure TCMS model, the communication between onboard systems and the data center is secured by the use of Securebox HSMs (sometimes called Secure gateway) as depicted in Figure 2. The HSMs secure the transmission of data and synchronization messages by implementing the Securebox architecture.

### 3.1. The Securebox Architecture

The security in TCMS was categorized in a domain called Industrial Control System (ICS) security which is covered in the IEC 62443 standard [20]. ICS security is slightly different from IT Security, as the failure of ICS security could cause physical damages that could lead to casualties and property losses. There is much research and best practice development that has been carried out in IT security. Next-generation firewalls [21], the machine-learning-based Intrusion Detection/Prevention System (IDS/IPS) [22], mobile Virtual Private Networks (VPN) [23], GSM-R to LTE-R [24], quantum cryptography [25], etc., can be implemented to secure the IT network.

However, there are specific concerns that we must address for ICS security, especially in the railway system. First, communication in TCMS needs reliable wireless communication for uninterrupted and low-latency network performance. The use of a VPN is not an option as the VPN will drive higher latency, and the train mobility requires the connection to be re-established each time the train moves to a different network coverage. The GSM-R can provide higher data rates according to eMLPP features in circuit-switched digital model connection between train and train control. However, it is prone to MITM attack as there is no end-to-end encryption in this protocol. This disadvantage also occurs in LTE-R even though it provides a higher data rate. GSM-R and LTE-R only provide data security between MS to BS. Thus, LTE-R has not been implemented in all rural areas along the railway, which makes it unsuitable for many TCMS. The advanced firewall and IDS/IPS can reactively protect the IT network with higher detection accuracy. However, they only inspect the incoming data in a single node but not the data transferred along the network. Thus, it is impossible to implement these devices in every node of the network or along the railway network. For data security, the advanced encryption algorithm research comes in the form of asymmetric and quantum cryptography. However, their higher computational cost is not suitable for the limited computational resource in the existing ICS environment in TCMS.

According to the ANSI/ISA 99 standard [26], the ICS security reference model is mapped in level 3 to level 1. Level 3 covers the operation management, which is the TCMS, level 2 is the supervisory control in the form of the monitoring function, and level 1 is the basic control of the on-board VCU in the train. Thus, we need a specific strategy to cope with the ICS security requirements [27]. This research proposes a Securebox architecture implemented in a Securebox HMS. To cover the scientific and industrial requirements, the architecture was developed in terms of the following considerations:

- Modular, which works as an add-on for an existing TCMS. Under this consideration, the TCMS and Securebox HSMs were efficiently developed and installed independently without adding more processing load to the TCMS or disturbing the train installation and operation.
- Open and independent, which was developed according to an open communication system standard and to be independent of the operating system. In this case, the application could run on any operating system and any data network.
- Secure, the security aspect was developed in terms of end-to-end data security. The cryptography algorithm encrypts plaintext data from the onboard train system and decrypts the encrypted data in the data server. The implemented cryptography algorithm is the most secure cryptography algorithm.
- As it would be a critical dedicated device, the development must meet the railway reliability standards; the applicable standards are EN 50155 [28] and EN 50121-4 [29], covering signaling and telecommunication apparatus installed inside the railway environment.

**Figure 3:**
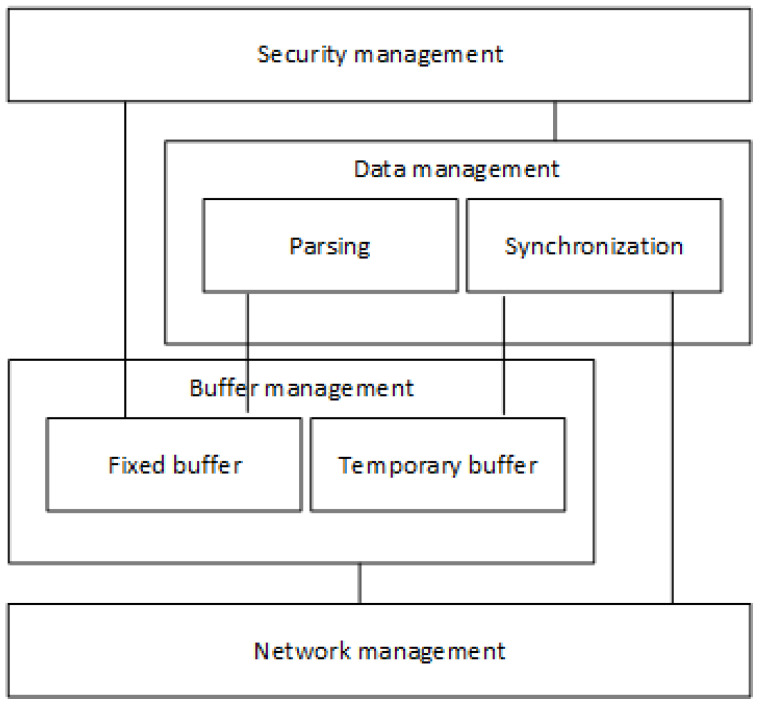
Securebox architecture.

The Securebox architecture is shown in Figure 3. It is composed of four main functions: network management, buffer management, data management, and security management. The main functions are described below, from the bottom to the top.

1. Network management

The network management function is responsible for successful communication between the Securebox HSMs and the data server. It handles the messages passing through the communication channel regardless of the transmission media. This function is responsible for network addressing, the logical link control protocol, and the medium access control protocol. It also handles the necessity of connection by virtual or logical links on the network. The virtual connection can be in the form of a peer-to-peer, client-server, or virtual network for a secure communication channel between a mobile VCU and the data server.

2. Buffer management

The buffer management function acts as a buffer for the received message from the VCU in the train and buffers the message, which it synchronizes and transmits to the data server. In the TCP/IP protocol suite, a data buffering function is developed in the application layer. The embedded synchronization algorithm is used to maintain data transmission from Securebox HSMs to the data server.

3. Data management

The data management was developed because the HSMs will be installed in a moving train. There was a consideration that if the train passed through a poorly connected region, the HSMs might be unable to contact the server, and hence would not send the data. Therefore, data management is responsible for the synchronization and data parsing function. Synchronization is a task to keep data integrity, ensuring that the data are successfully transmitted to the data server even if the train passes through a poorly connected region. Data parsing is a task to cleanse the data from any character that was not needed and split the data according to the data separator. The data separator is a character that marks the end of a data entry and the beginning of the following data entry.

4. Security management

Cryptography is a function responsible for data confidentiality and authenticity. Cryptography is used to preserve confidentiality by encrypting the plaintext data from the VCU before it is sent to the data server. In this case, the data was encrypted to non-readable encrypted data to ensure that any attacker could not read the plain data without the cryptographic key. The user access control was implemented at the user and hardware level to meet the authenticity requirement. Security management must ensure the security of the operating system and its application as well.

### 3.2. The Securebox HSM Design

To fulfill the modular requirements, the Securebox HSM was designed to work independently, enhancing the existing process without adding more processing load to the existing device. In testing, the existing onboard train VCU used a Programmable Logic Control (PLC)-based Selectron CPU 833-TG, which was already set to send data using File Transfer Protocol (FTP). Thus, the developed HSM must act as FTP server for the VCU and as a computer client for the data server. In this scheme, the Securebox HSM must maintain all data transmission even if any bad or blank spot connections occur along the railway. The HSM then sends the data to the data center via Hypertext Transfer Protocol (HTTP) using a one session TCP handshake for each packet.

**Figure 4:**
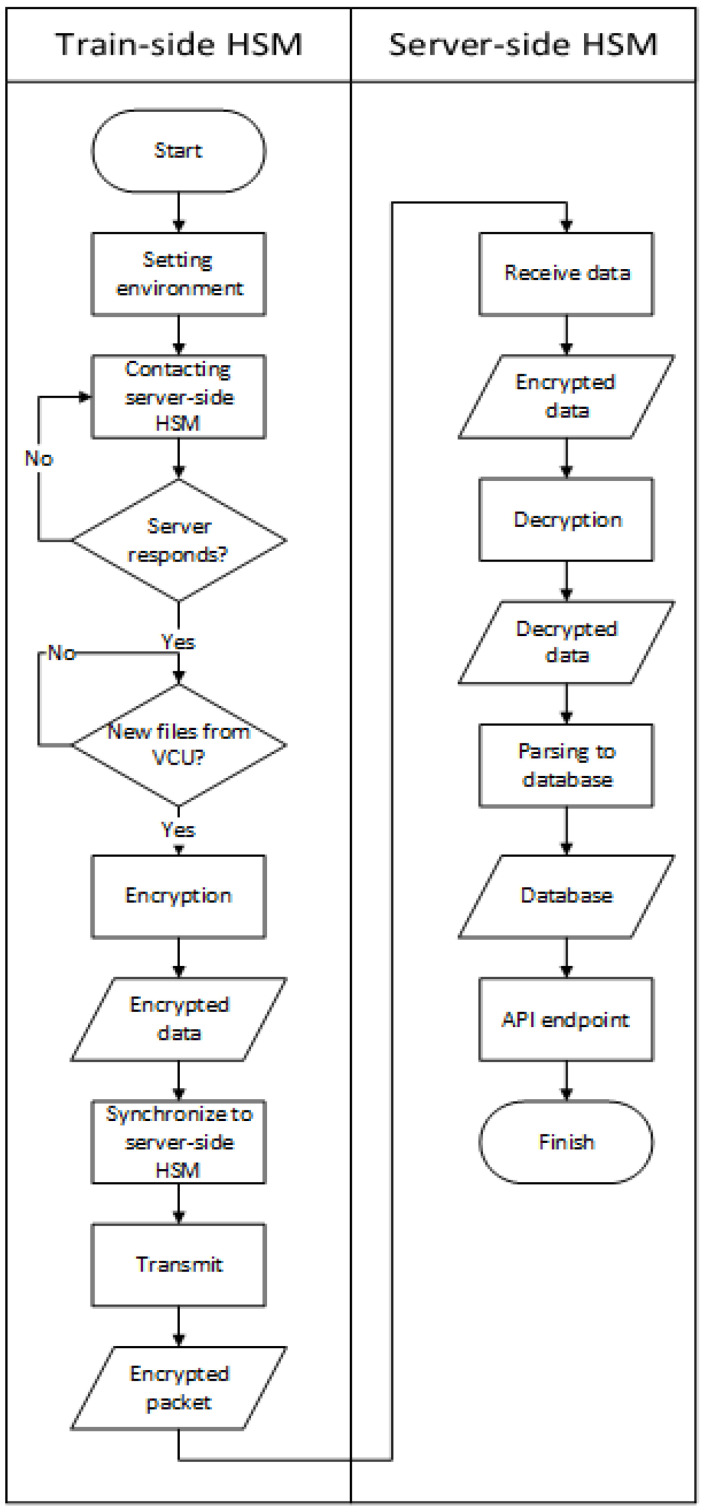
Securebox HSMs functional flow.

The Securebox HSMs were developed based on x86 computer architecture [30] and a standard TCP/IP protocol [31] for communication protocols. The x86 computer architecture was chosen because major TCMS devices have been developed to support various computer platforms. Likewise, the TCP/IP protocol standard was chosen because almost all TCMS devices support the communication protocol. The architecture implementation is done for each function layer independently. The HSMs were set up in two modes. The first is the train-side HSM in the onboard train system and the other is a server-side HSM. The workflow of the Securebox HSMs is depicted as a flowchart in Figure 4.

**Figure 5:**
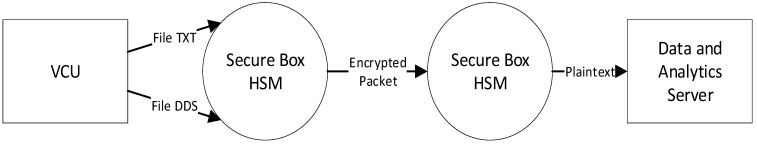
Securebox HSM DFD Level 0.

**Figure 6:**
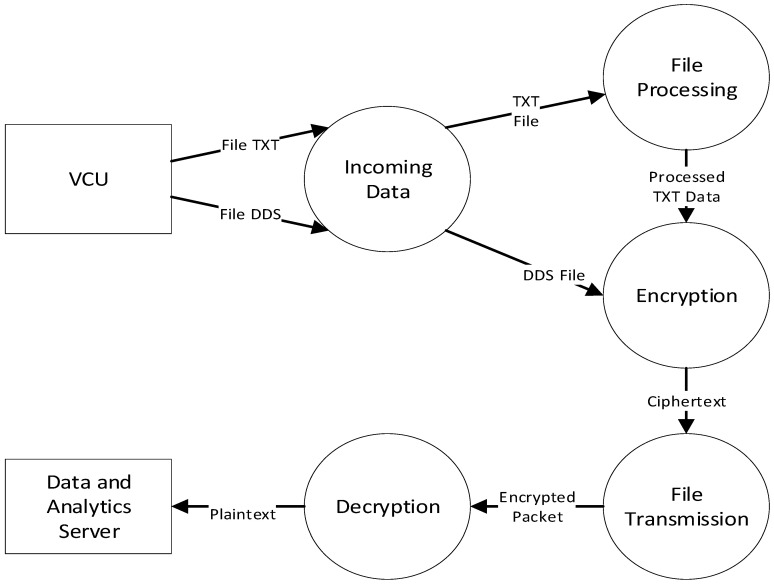
Securebox HSM DFD Level 1.

**Figure 7:**
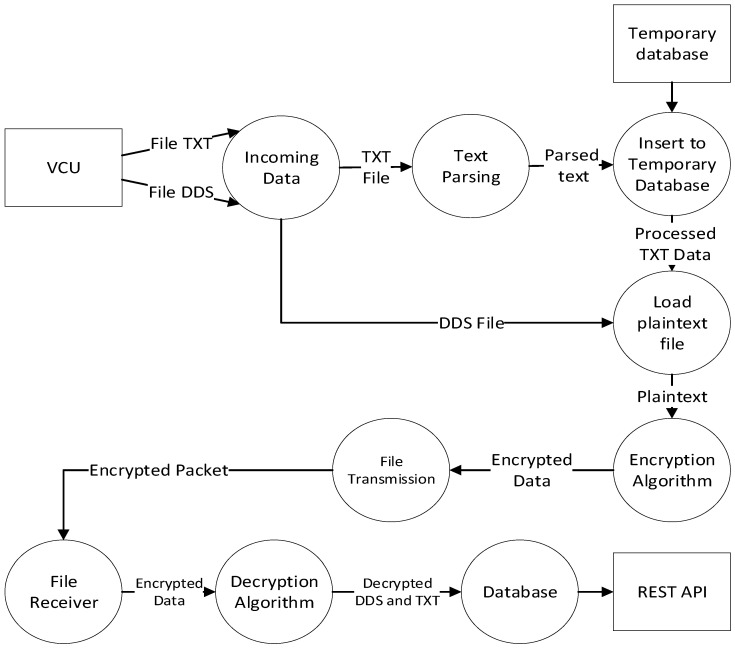
Securebox HSM DFD Level 2.

In the train-side HSM, the VCU sends two kinds of data, which are *.txt and *.dds files, to the Securebox. The *.txt file contains routinely plaintext data, which are sent periodically to the Securebox (in this case, taking 5 s). The *.dds are plaintext data, triggered on request or by any registered events. The Data Flow Diagram (DFD) for a TCMS which implements Securebox architecture can be seen in Figure 5 for DFD level 0, in Figure 6 for DFD level 1, and in Figure 7 for DFD level 2.

### 3.3. The Secure TCMS Setup

The train-side Securebox HSM was installed on a diesel-electric train produced by the Indonesian Rolling Stock Industry, and the server-side Securebox HSM was installed in the colocation server in the Telkom University data center. The hardware was a certified railway computer with an x86 64-bit-based i7-7600U computing architecture with 16 Mb DDR4 RAM. The computing architecture had the sufficient computing power to run the Securebox application and withstand harsh railway conditions. The prototype was connected to the VCU using a railway-standard ethernet cable and TCP/IP protocol. The Securebox HSMs ran in two algorithms, 3DES and AES, in CBC (Code Block Chain) block cipher mode. The testing was intended to measure the performance of our architecture compared to the existing state-of-the-art [7]. However, in this test, the 3DES was run in a x86 computing platform, not in the IoT-based TCMS. because the Securebox must comply with EN 50155, EN 50121-4, and IEC 61373 device standards.

The VCU collected real-time data from sensor devices in the train and sent the data every 5 s as a *.text file format to the train-side Securebox HSM. The HSM then connected to the public network through a 4G-LTE modem on the train. The test was done from Bandung to Singaparna station on Java Island in Indonesia. The trip took almost an hour and 15 min along urban, suburban, and rural conditions to evaluate the performance of the secure TCMS. However, the *.dds data was sent just once after the trip finished, because the data were an aggregate of all sensor data along the trip. The longer the journey, the greater the amount of data that will be sent .which is much greater than the *.txt data. For integrity and quality of service purposes, the data were set to be sent only one time after the trip finished.

The secure TCMS setup was done in two sites, which were an onboard train system setup and a data server setup. The setup details follow.

1. Onboard train system setup

In the onboard train system setup, the sensor network from the train devices sensors was connected to a Remote I/O Module (RIOM) in each train carriage and connected to one VCU located in the train control room. The VCU data output was then transmitted to an HSM via ethernet protocols. In the HSM, the Securebox application was installed and programmed according to the Securebox architecture. The application handled the functional architecture as below.

- Network management

The networking function used two Network Interfaces Cards (NIC). The first was the NIC-facing VCU, and the second faced the 4G modem device. It utilized ethernet protocol, as it is broadly used in the TCMS system. A Shielded Twisted Pair cable was chosen to be used in ethernet transmission media, as it proved to be more resistant to electromagnetic interference than wireless media used in a wireless protocol. Thus, it already complied with the existing infrastructure in the TCMS and did not interfere with the TCMS operation.

b. Buffer management

The received data from VCU were obtained and buffered in an FTP server. After the file was processed for data management and security management, the data were sent to the data server via HTTP with a specific decryption port address. When the synchronization function meets any unsuccessful HTTP transmission condition, the data will be kept in the buffer and are sent when the connection is available. This function is also responsible for keeping data integrity by the use of a hash function for every datum.

c. Data management

Securebox acted as an FTP server for the VCU. After receiving the file, the synchronization and parsing functions were developed using the Go language. Go was designed for multicore computers. Therefore, it can maximize the performance of a multicore CPU [32]. The synchronization manages the connection to the data server and interacts with the data buffer. If the connection is successful, then the data are deleted from the buffer, and when the connection is lost, the data remain in the buffer until the connection is successful. The data from the VCU was sent via HTTP by a one session TCP handshake for each packet. The parsing function separated data according to the specific data sensor recorded in the VCU.

d. Security management

The Securebox implemented AES and 3DES symmetric cryptography algorithms, which are developed in the Golang language. AES was used as the main cryptography algorithm, and 3DES was used as a comparison based on research in [7]. The symmetric algorithm was used as it is a lightweight algorithm and there was no need for key exchange distribution in the implementation. With the symmetric algorithm, the key setup is done once in the Securebox registration process in the data server. The symmetric key algorithm also provides broader choices for the algorithm to use. In this implementation, the AES algorithm in Cipher Block Chain (CBC) mode is used as one standard algorithm for modern cryptography [33]. When it is configured as train-side HSMs, then its application works in encryption mode, and then the application works in decryption mode when configured as server-side HSM.

2. Data server setup

The data server consisted of a server-side HSM and a data server. The security management function in the server-side HSM decrypts the ciphertext to obtain the plaintext. The plaintext data are then saved according to the data parsing position in a database. The database is crucial because it manages the data in the data server and is visually displayed in the monitoring function. The data in the database will proceed further for analytical purposes. The data server manages the plaintext received from the server-side HSM and acts as the database function and the monitoring function.

- Database function

The database function is used as a repository for plaintext data from server-side HSM. In this test, PostgreSQL was used as it is a high-reliability database platform and can run on almost all operating systems [34]. The database consists of one table. The table contains Epoch time, which includes the time when the data are generated by the VCU, and 12 parameters, v1 to v12, which consist of the data parameters generated by the VCU. The data are generated as a binary number. Here, the data are saved in plaintext since the data server handles the security aspect. The further analytical process of the data is not discussed in this paper as it will be discussed in a further report.

b. Monitoring function

The monitoring function is a function where all sensor status data in TCMS are displayed. To receive the data, the monitoring function connects to the database. The connection can be in the form of a database query or by API. For security reasons, API is preferable since it lessens the probability of SQL injection attacks [35]. API itself can be either PULL or PUSH [36]. The monitoring was done on a web-based platform. To connect to the database, a pulled API has been developed where the monitoring application could request data from the database according to its need and according to what data are served by the API endpoint.

### 3.4. Validation

1. Blackbox Testing

In the blackbox testing, the system was analyzed based on application details which are the functions that exist in the application. This test did not inspect and test the source code of the program. It analyzed the function flow of the system to suit the business processes of the Securebox architecture, which are:

- Data connection. The data connection function was tested in three scenarios using an internet connection and a TCP connection. The result can be seen in Table 1.
- Parsing data. The parsing data function validation used several scenarios: incoming data to Securebox HSMs, the data separation based on file extension, the contents of the *.txt file parsed by the parsing module, and the contents of the *.txt file entering the temporary buffer. The validation result is shown in Table 2.
- Data synchronization. The data synchronization function was validated in three scenarios: the HSM and data server contact, sending synchronization messages from HSM, and replying to synchronization messages. These scenarios are very important due to the possibility of a lost connection along the train trip. The synchronization validation output is shown in Table 3.
- Data encryption and transmission. The data encryption and transmission function were evaluated in several scenarios, which were: detect the *.dds file, encrypt the *.dds file, send *.dds encrypted data file, load the content of *.txt file, encrypt the content of *.txt file, and send the encrypted *.txt file. The result is shown in Table 4.
- Data receiving and decryption. The data receiving and decryption function is done in the data server. Three scenarios were used to evaluate the output: receiving the ciphertext (encrypted data), decrypting the ciphertext, and detecting the plaintext output. The validation result is shown in Table 5.

From the Blackbox testing, the secure TCMS was successfully implemented and met all the software development life cycle requirements. From this behavioral test, all the required functions in the prototype worked and were integrated properly in all testing scenarios. The Securebox HSM connected and sent all data from the VCU, secured, and transmitted securely to the data server. The secured data also was successfully received and stored in the data server database.

2. Whitebox Testing

In whitebox testing, several scenarios were deployed to verify the actual output of the system modules’ program code.

- Data were sent from the VCU to the train-side Securebox HSM by testing the specific programming code to support the HSM FTP server testing. The scenarios were: a *.dds file sent from VCU to HSM buffer management, a *.txt file sent from VCU to HSM buffer management and parsing function, and other files sent to HSM file folder but not proceeding further. The result is shown in Table 6.
- Establish connection. It was done by observing the specific program code to support secured connection testing from the onboard train system to the data server. The result is shown in Table 7.
- Data Synchronization. Data synchronization testing was done by testing specific programs to support integrity functionality. The testing used four scenarios, which were: sending synchronization data to the data server whether the connection was available, synchronization data to the data server with no connection available, synchronization data to the data server after the connection was lost for 1 min, and synchronization data to the data server after a connection was lost for 5 min. The scenario and results are available in Table 8.
- Encrypt and transmit data. The encryption and transmission testing was done in the server-side HSM to ensure the programming code met the confidentiality requirement. Several scenarios were tested by encrypting the *.dds file, encrypting the *.txt file, and sending data when the onboard train system was connected to the 4G network. The result is shown in Table 9.
- Data decryption. The decryption testing was done in the server-side HSM, right after the data were received. The testing analyzed the program code to meet the required output for the decryption of the *.txt and *.dds files. The output can be seen in Table 10.

From the whitebox testing, the program code of the Securebox application gave all the expected results. The whitebox analysis showed that all the module and the program code in the module were checked and analyzed to produce the expected output. There was no malfunction or wrong programming code, which can produce a system error.

## 4. Discussion

### 4.1. Performance Analysis

System performance analysis aimed to evaluate the use of Securebox HSMs as a security module added to the secure TCMS, especially to analyze how the added module affected the secure TCMS’s overall performance. The testing measured the performance of the HSMs in encryption time, transmission time, display time, and security. The encryption time is the time needed for a file to be encrypted by the encryption algorithm in the HSMs computing platform. Transmission time was determined from the time the file is generated by the VCU, file detection by the file detector, data encryption, and data to the data server until the server received the data. The data display time was determined by calculating the decrypt processing time, inputting the data to the database, and then loaded to the web interface in the data server platform.

1. Encryption time

The time required for the train-side Securebox HSM to perform encryption varies according to the size of the file. In the *.txt file, each file is generated by the VCU in a fixed 360 bytes size. This file contains 60 data entries that were generated by the VCU every 5 s. The encryption time for the *.txt file was 4 ms for AES and 6.2 ms for 3DES on average which means AES provides 55% faster.

The encryption algorithm complexity heavily influences the encrypted time. The AES algorithm performs key generation, AddRoundKey, SubBytes, ShiftRows, and MixColumns process for 10 rounds for 128-bit keys encryption. The AES and 3DES algorithms were run in the block cipher. It means the plaintext was encrypted block by block in fixed size block data. The block-based encryption in AES performs encryption for every 128-bit block of data input. Thus, overall encryption time varies depending on the size of the encrypted file, as it affects the number of the encryption process. The decryption time to decrypt each ciphertext is also unnoticeable around 4 ms, the same as the encryption time.

The encryption time for the 3DES algorithm is higher than AES, as the 3DES works in a 64-bit block cipher, which produces a double number of block plaintext to be encrypted. The Feistel network for the encrypt-decrypt-encrypt process was also raised to 48 rounds for each block. It ended up with a higher encryption time than the AES, especially for the bigger file size.

2. Transmission time

**Figure 8:**
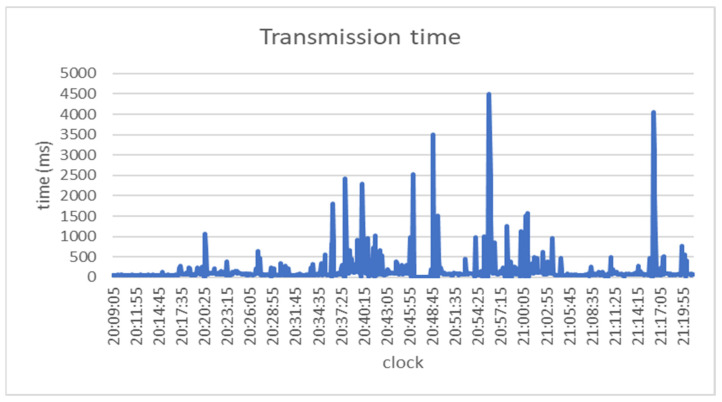
Transmission time.

The transmission time with or without the added Securebox HSMs was the same on average. From the transmission time test result, the median for transmitting 520-bit *.txt data was 77.04 ms. The median was used as it is a robust statistical analysis that can withstand the outlier. The transmission time is shown in Figure 8.

The buffer management worked properly as there was no data loss at the data level. The high spikes of the transmission time are highly connected due to the signal strength or in a handover situation. The data transmission time variance was caused by the 4G signal strength variance captured by the modem along the train journey. It affected the transmission performance as it caused the data transmission to fail, and the data was kept in the synchronization buffer until it was successfully transmitted. For the transmission of *.txt data, the transmission time with or without the added Securebox HSMs was the same on average, as the encryption took only 4 ms on average.

3. Display time

The display time analysis was conducted on the *.txt file only, as the *.dds data were encoded and needed an additional decoded process to be inputted and processed in the monitoring system. The *.txt file was not encoded data. Thus, it entered the decryption process once received in the buffer of the data server.

From the analysis, the display time was highly affected by the database size. The larger database size will result in a higher display time. The average time required for the received *.txt encrypted file to reach the user interface in the monitoring display was 391 ms. This result is affected by the additional time needed to query all data from the database to be displayed in the monitoring view of the database contents.

These time performances constitute the delay parameter in a secure TCMS. This parameter is important due to the data-driven behaviour of the TCMS, which must maintain the continuity and sustainability of the running operation. From our result, the encryption and display time is deterministic. The most influential delay parameter is the transmission time, which is affected by the wireless transmission environment or when the network is under active attack. In the performance tests, the transmission performance was highly affected by the unpredictable underlying wireless transmission environment. However, the average 4.5 s time variance still managed to produce no data loss at the data level due to the use of buffering management in the Securebox architecture. Thus, it still assured the continuity and sustainability of the secure TCMS, which in this case sent data every 5 s. In case of the synchronization fail, the data are kept in the HSM buffer and will send after the synchronization success. The stochastic behaviour of the transmission time may be heuristically analyzed to determine the optimal transmission buffer size.

4. Computing performance

**Figure 9:**
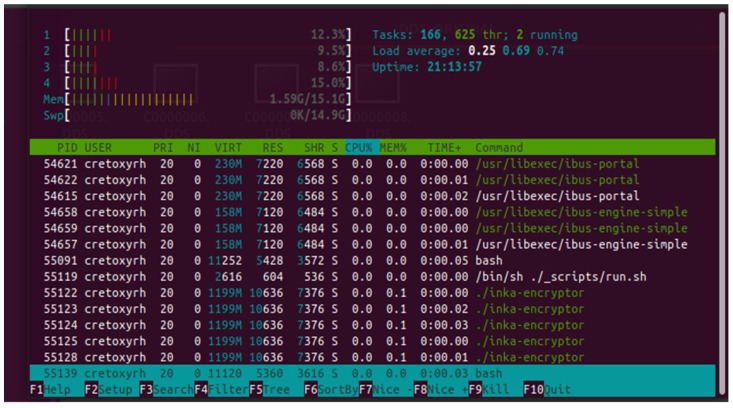
Computing performance.

From the computing performance, the increase in average processor load, while the encrypt and delivery daemons are running, is unnoticeable (0% on average) in x86 i5 64-bit processor architecture environments. Further increase in memory load is also not visible (0.1% on average). In the captured task manager image in Figure 9, the most significant burden affecting the computing performance was from the user interface in the Securebox HSMs for development purposes.

### 4.2. Security Analysis

From the security aspect, confidentiality, integrity, and authenticity must be preserved. The access control from application to hardware authentication preserved the authenticity aspect in the secure TCMS. Starting with operating system security, the Securebox application was run in a virtual OS environment in a specified OS user access control to disable another application installation, thus preventing OS-level malware and rootkit attacks. The specific hardware physics and logic address registration is done before the HSM is located on the train. The application was also developed with specified port knocking and port numbers for application-level authentication. This access control scheme protects the secure TCMS from MITM, network malware injection, malicious node injection, and further firmware attacks.

**Figure 10:**
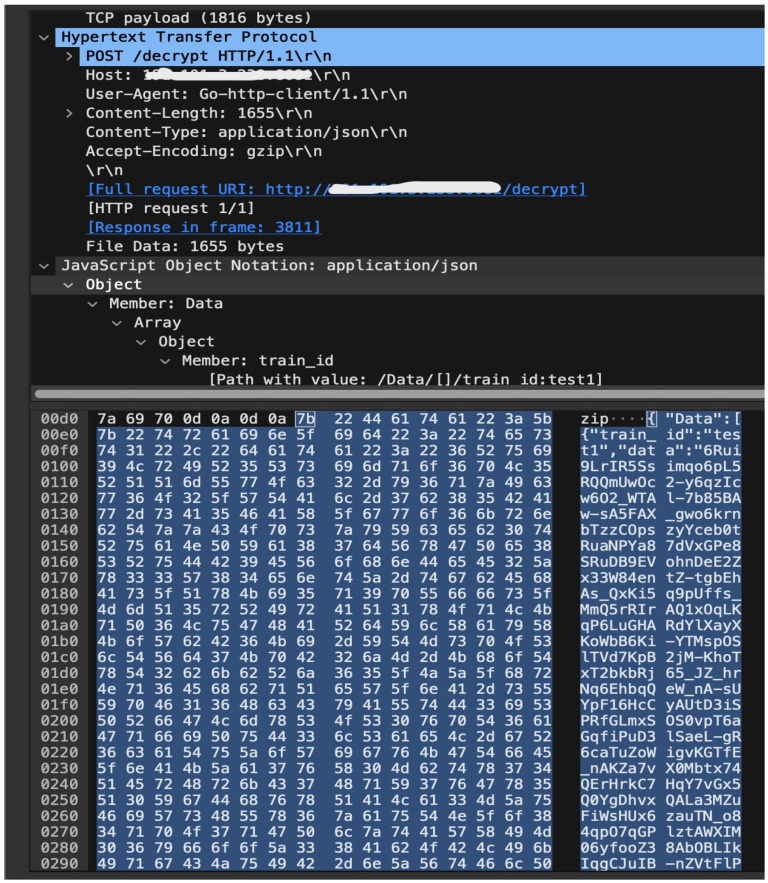
Secured unreadable data from eavesdrop attack on secure TCMS.

A MITM attack simulation was carried out in the confidentiality and integrity test by disabling the access control. The simulation was carried out when the train-side Securebox HSM had encrypted data. It was assumed that the attacker had access to either the network of the train or the data center, therefore they could eavesdrop on the connection. The results based on the security test found that the HSM can encrypt well, and the encrypted data obtained in the eavesdropping attack was much different from the plaintext sent. Figure 10 shows the unreadable ciphertext of the eavesdropping attack on the secure TCMS.

**Figure 11:**
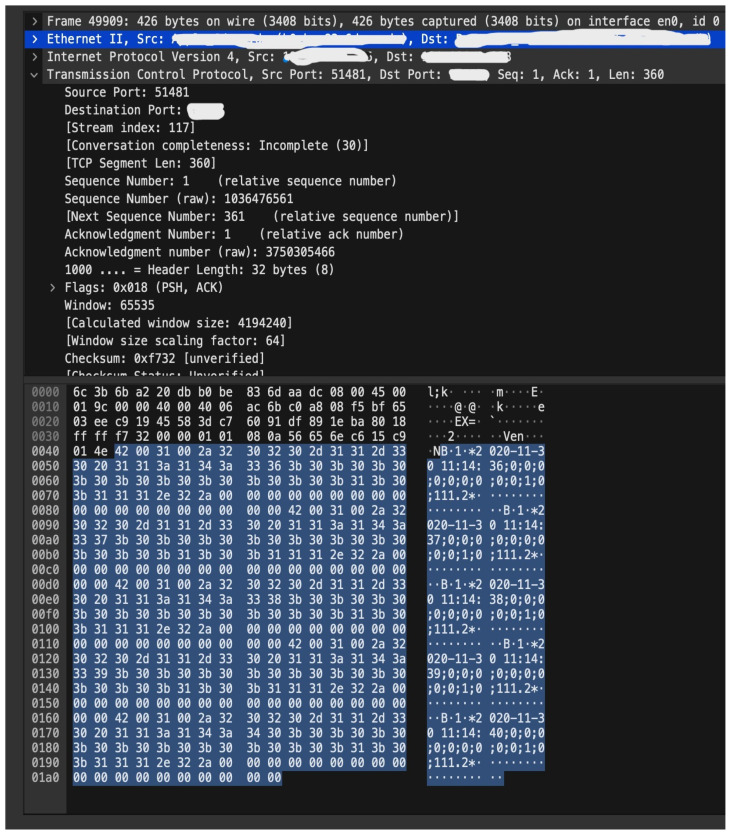
Readable data from eavesdrop attack on GSM-R via unsecured network, and the data could be understood by malicious party.

The MITM simulation also made a data modification and injection attack. However, this kind of attack did not breach the integrity aspect as Securebox application implemented authentication and integrity checking in the data transmission. Thus, it can mitigate the risk of repetition, insertion, corruption, and masquerade data. The MITM attack scheme is done in a node in front of the server-side HSM. Figure 11 shows the readable plaintext captured from traditional FTP-based TCMS using GSM-R only.

From the cryptoanalysis aspect, the Securebox HSM implements the AES algorithm in a CBC operation mode in Securebox HSM, in which the encryption is performed in a chaining manner. The output of an AES block becomes the input to the next AES block. This operation mode aims to withstand brute force attacks, considering that the same character can be output from different encrypted data. The output file shows that the same input character (character ‘0’) can be a very different output. This characteristic of the AES-CBC algorithm has made the system more secure, as it makes it more difficult for attackers to acquire the plaintext, even though the characters in the plaintext are the same. The further comparison of the cryptanalysis can be found in [37], especially for large amounts of data with a single key.

In case of an active tampering attack, the transmitted data can be captured, deleted, delayed, reordered, and modified by the attacker. It directly affects the time performance, as the transmission time will be higher. In this case, the integrity checking function in Securebox HSM manages the delayed, re-ordered, and modified data attack, by its synchronization and buffering function. In the server-side Securebox HSM, if the received data are not the intended data, then it will discard these and request the intended data.

However, as the secure TCMS sends the data on a public network, the system is still prone to link-based DDoS and deletion data attacks. Anyone in the network can capture and drop the data or flood the network, which leads to data loss due to the unavailable bandwidth. In this case, the Securebox architecture only protects the data but not the network. The data may still be lost but the HSM can detect the active attack. This can be done by setting the data continuity assurance time parameter. This parameter indicates the maximum time between two consecutive displayed data on the monitor screen. If the intended data are lost for more than the continuity assurance time threshold, then it may indicate that there is an anomaly in the network, which affects the train safety aspect. From the fail-safe principles of train systems, further control may be deployed such as an emergency braking for the related train.

However, it only protects the server but not the data in networks. For further study, when the computational resource of the ICS is not an issue, it is possible to implement Collaborative Intrusion Detection Network (CIDN) [38], by implementing collaborative IDS/IPS between the onboard train and data center. This can proactively detect a traffic anomaly in the network. The specific prevention action for the fail-safe principle of the train system can also be studied further.

## 5. Conclusions

In this study, secure TCMS has been designed in the Securebox architecture. The Securebox architecture consists of four main functions: network management, buffer management, data management, and security management. Based on the black box and white box testing methods, the Securebox application can perform the main functions that have been designed. Based on the results of Securebox HSMs tests carried out on the diesel-electric train produced by the Indonesian Rolling Stock Industry, the Securebox HSMs have not degraded the TCMS performance. The average transmission time is the same as without the HSMs, and the encryption time is unnoticeable in a fraction of a millisecond. The use of the AES algorithm provides a 55% faster encryption time than 3DES. It wards the TCMS from the risk of repetition, insertion, corruption, and masquerade data from the MITM attack. It also proved to provide end-to-end data protection compared to the use of GSM-R MS to BS only protection. For further study, it is possible to implement CIDN, by implementing collaborative IDS/IPS between the onboard train and the data center to proactively detect traffic anomaly in the network. The specific prevention action for the fail-safe principle of the train system can also be studied further.

## Figures and Tables

**Table 1:** Connection Testing.

Scenario	Planned Output	Actual Output
Securebox HSM internet connection	HSM is connected to the internet network via an 4G modem	HSM is connected to the internet network via an 4G modem

**Table 2:** Parsing data testing.

Scenario	Planned Output	Actual Output
Data go to Securebox HSM	Data received by FTP	Data received by FTP
Data separated by file extension.	Data separated by file extension	Incoming data are separated according to the file extension. *.txt files go to the TXT folder, *.dds files go to the DDS folder, and other file types are rejected
Data are treated according to the file extension	Files with *.dds format go directly to the encryption module. Files with *.txt format go to the parsing module	Files with *.dds format go directly to the encryption module. Files with *.txt format go to the parsing module
The parsing module parses the contents of the *.txt file	The parsing module can separate the contents of *.txt files	The parsing module separates the contents of the *.txt file according to a predetermined limiter, namely semicolon (;)
The contents of the *.txt file enter the temporary database	The contents of the parsed *.txt files are entered into a temporary database	The contents of the parsed *.txt files are entered into a temporary database

**Table 3:** Data synchronization functional testing.

Scenario	Planned Output	Actual Output
Contact between the Securebox HSM and the data server	Securebox HSM can contact the data server	Securebox HSM can contact the data server
Sending synchronization messages from HSM	Securebox HSM can send synchronization messages to receiving data server	HSM can send synchronization messages to receiving data server
Reply synchronization message	The data server can reply to synchronization messages from HSM	The data server can respond to synchronization messages from HSM

**Table 4:** Data encryption and transmission testing.

Scenario	Planned Output	Actual Output
Detect the *.dds file	*.dds data detected in the DDS folder	*.dds data detected in the DDS folder
Encrypt the *.dds file	*.dds files can be encrypted by the encryption module into a ciphertext (encrypted data)	*.dds files can be encrypted by the encryption module into a ciphertext (encrypted data)
Sending the *.dds file	The *.dds ciphertext file (encrypted data) is sent from the HSM to the IP address of the receiving data server	The *.dds ciphertext file (encrypted data) is sent from the HSM to the IP address of the receiving data server
Load the *.txt file content	The content of the *.txt file from the database is loaded into the encryption module	The content of the *.txt file from the database is loaded into the encryption module
Encrypt the *.txt file content	The encryption module encrypts the contents of the *.txt file of the database	The encryption module encrypts the contents of the *.txt file of the database
Sending the *.txt file content	The ciphertext (encrypted data) of the *.txt file content is sent from the HSM to the IP address of the receiving data server	The ciphertext (encrypted data) of the *.txt file content is sent from the HSM to the IP address of the receiving data server.

**Table 5:** Data receiving and decryption testing.

Scenario	Planned Output	Actual Output
Receiving encrypted packet	Encrypted packet received by the data server	Encrypted packet received by the data server
Receiving ciphertext (encrypted data)	Ciphertext (encrypted data) received by the data server	Ciphertext (encrypted data) received by the data server
Ciphertext (encrypted data) decryption	The decryption module decrypts the ciphertext (encrypted data) into plaintext	The decryption module decrypts the ciphertext (encrypted data) into plaintext
Plaintext output detection	The plaintext is placed according to the extension	The plaintext is placed according to the extension. Plaintext *.dds is placed in the DDS folder in the form of a binary file. Plaintext *.txt file results are placed in the plaintext database.

**Table 6:** Securebox HSM FTP server testing.

Scenario	Input	Expected Output	Actual Output
*.dds file sent from VCU to Securebox HSM	File *.dds	The ciphertext (encrypted data)	The *.dds file goes to FTP, is detected as a *.dds file, and goes to the encryption module. The encryption module encrypts the *.dds file into a ciphertext
*.txt file sent from VCU to Securebox HSM	File *.txt	The ciphertext (encrypted data)	The *.txt file goes to FTP, is detected as a *.txt file, and goes to the data parsing module. From the parsing module, the data file entered the database then runs a load entry query and performs encryption
Other files sent to Securebox HSM	Other files	Fail	The file is not recognized by the application and is immediately deleted from the Securebox.HSM

**Table 7:** Securebox HSM establishes connection testing.

Scenario	Input	Expected Output	Actual Output
Securebox HSM establishes a connection to the data server	Data server IP address	Receive reply	Receive reply TCP 200 from the data server to HSM

**Table 8:** Data synchronization testing.

Scenario	Input	Expected Output	Actual Output
Securebox HSM sends synchronization data with the connection condition connected	Synchronization data bit	Reply from the data server	The Securebox HSM responds by running the data transfer module
Securebox HSM sends synchronization data with no connection condition	Synchronization data bit	No reply	No reply from the data server. The HSM stores the undelivered data in a buffer
Securebox HSM sends synchronization data after the connection is lost for 1 min	Synchronization data bit	Reply from the data server	The HSM runs the data transfer module and stored in the buffer for 1 min
Securebox HSM sends synchronization data after a connection is lost for 5 min	Synchronization data bit	Reply from the data server	The HSM runs the data transfer module and stored in the buffer for 5 min

**Table 9:** Encryption and transmission testing.

Scenario	Input	Expected Output	Actual Output
Encrypt the *.dds file	File *.dds	The ciphertext	The ciphertext (encrypted data)
Encrypt the *.txt file	Database entry of the contents of the *.txt file	The ciphertext	The ciphertext (encrypted data)
Transmits when the HSMs are connected to the internet	The ciphertext	The ciphertext sent from HSM to the data server	The ciphertext sent from HSM to the data server
Transmits when the HSM is not connected to the internet	Fail	The ciphertext is not sent and remains in the buffer	The ciphertext is not sent and remains in the buffer

**Table 10:** Decryption testing.

Scenario	Input	Expected Output	Actual Output
Decrypt encrypted data	Ciphertext (encrypted data) generated from *.dds files	File *.dds	File *.dds
Decrypt encrypted data	Ciphertext (encrypted data) generated from database entry of *.txt file data	Database entry on the data server	Database entry on the data server

## Data Availability

Data available on request.
